# An OCT Study of Anterior Nodular Episcleritis and Scleritis

**DOI:** 10.1155/2017/5742673

**Published:** 2017-02-28

**Authors:** Christos Christakopoulos

**Affiliations:** Department of Ophthalmology, Zealand University Hospital, Næstved, Denmark

## Abstract

Anterior scleritis and episcleritis are a well-known presentation in tuberculosis. The case of a female patient with presumed tuberculous anterior scleritis and episcleritis is discussed in this article. Anterior segment OCT was efficient in diagnosis and evaluation of the therapeutic outcome. Antituberculosis chemotherapy was sufficient to achieve clinical remission.

## 1. Introduction

Tuberculous scleritis is rare [1–3]. Commonly it presents as anterior nodular scleritis, which can be caused either by direct inoculation [4] or by hematogenous spread of* Mycobacterium tuberculosis* (*M. tuberculosis*) [1]. The characteristic nodular inflammatory lesions can be detected by slit-lamp biomicroscopy but assessment of their vertical extension is difficult. However, recently, it was reported that optical coherence tomography (OCT) of the anterior segment can be used in diagnosing anterior scleritis and episcleritis [5], and here I report the OCT characteristics and changes during treatment of a nodular eye wall lesion in a patient infected with* M. tuberculosis*.

## 2. Case Presentation

A 52-year-old female of West African origin was referred to the Department of Ophthalmology, Zealand University Hospital. The patient had for the last 5 years been treated with Dexamethasone (1 mg/ml) eye drops (Maxidex, Alcon) on an on-off basis because of a red right eye (RE). Local therapy was not helpful and the patient had discontinued the treatment weeks before admission to our department.

**Figure 1:**
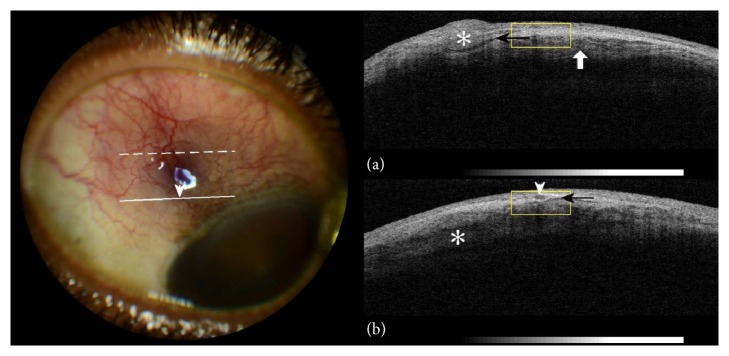
On the left side: colour photography of the right eyeball; congestion of the superficial blood vessels and dilation of the episcleral vessels (arrowhead). On the right side: (a) anterior segment OCT (dashed line on eyeball photography). Nodular episcleral thickening (asterisk), subepiscleral fluid level (black arrow), and intralamellar scleral oedema (white arrow). (b) Anterior segment OCT (solid line on eyeball photography). Transverse section of dilated episcleral vessel (arrowhead) corresponding to relevant location on eyeball photography. Subepiscleral fluid level (black arrow) and intralamellar scleral oedema (asterisk).

**Figure 2:**
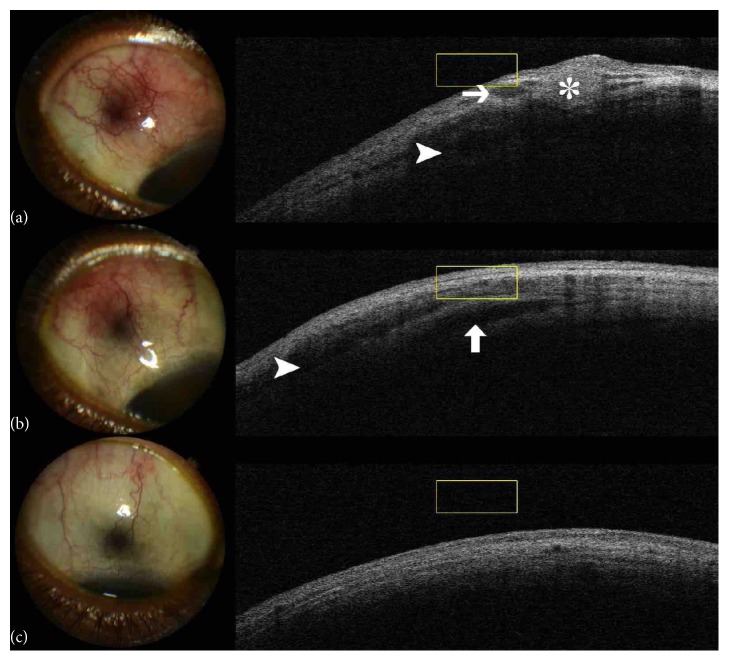
(a) RE, superotemporal sector hyperaemia, episcleral nodule (asterisk), subepiscleral oedema (arrow), and scleral oedema (arrowhead). (b) RE, after antituberculous chemotherapy initiation, hyperaemia subsided and nodules have disappeared but subepiscleral (arrowhead) and scleral oedema persist (arrow). (c) RE, after termination of therapy, no hyperaemia is present, and no oedema can be detected in the scleral or episcleral layers adjacent to previously detectable lesion.

On admission the patient experienced a persistent sense of ocular congestion and pain in RE. Left eye (LE) was asymptomatic. Visual acuity was 20/40 in RE and 20/15 in the left eye. Slit-lamp examination showed violaceous ocular injection around a subconjunctival nodular thickening at 4 mm from limbus and vessel dilation of both the superficial and deep vascular plexuses (Figures 1 and 2(a)).

Anterior segment OCT (spectral-domain OCT, 3D OCT-2000; Topcon Corp, Tokyo, Japan) at the center of the hyperemic lesion revealed hyporeflective spaces and hyperreflective nodules on the episcleral tissue band (Figures 1(a), 1(b), and 2(a)). Hyporeflective spaces bisecting the scleral lamellae were also apparent (Figures 1 and 2(a)). These findings were consistent with nodular episcleritis and anterior scleritis in the RE.

Ultrasound B-scan and dilated fundus biomicroscopy demonstrated no signs of posterior scleritis.

Swabs from conjunctiva for* M. tuberculosis* PCR analysis returned negative.

Comprehensive serologic evaluation including QuantiFERON-TB Gold, venereal disease research laboratory (VDRL), rapid plasma reagin (RPR), antineutrophilic cytoplasmic antibodies (ANCA), and angiotensin converting enzyme (ACE) was performed. The patient tested positive on Quantiferon assay. Chest radiography showed no pathological changes. In addition, further medical work-up performed at the Department of Infectious Diseases, Zealand University Hospital, revealed no other sites of tuberculosis.

Due to suspicion of tuberculous scleritis and episcleritis, antituberculosis chemotherapy was initiated, comprising Ethambutol, Pyrazinamide, Isoniazid, and Rifampicin. On follow-up the patient reported immediate improvement in ocular symptoms. Ocular examination with OCT confirmed reduction in the size of the hyperreflective nodules in the episcleral layer band but enduring hyporeflective intrascleral spaces (Figure 2(b)).

After 4 months of initial treatment the patient continued with Isoniazid and Rifampicin for another 2 months. On examination upon conclusion of antimycobacterial chemotherapy the patient reported no ocular discomfort and ocular examination demonstrated total remission of the hyperemia (Figure 2(c)) and elimination of the episcleral and the scleral lesions.

## 3. Discussion

Tuberculous scleritis is a well-documented condition but tuberculous nodular episcleritis has only sparsely been described before [6]. A report on tuberculous sclerokeratitis suggested the usefulness of OCT in anterior segment disease evaluation [7]. The presence of episcleral and scleral component at the same location suggests initial development of nodular episcleritis, probably representing the primary lesion, and subsequent involvement of the sclera as manifested by the intralamellar hyporeflective spaces on OCT (Figures 1(b) and 2(b)). Current research findings support an association of the latter scleral morphology on OCT with clinical scleritis [5].

Another report has previously emphasized the clinical suspicion of tuberculosis in cases of scleritis unresponsive to anti-inflammatory medications [8]. In the present case the positive Quantiferon assay [9] along with patient origin from a country of high prevalence [10] raised the clinical suspicion of tuberculosis. Quantiferon assay has been applied before in the diagnostic assessment of presumed tuberculous scleritis primarily due to the high specificity of this method [11]. Additional data indicate that it may exhibit better sensitivity than Mantoux test in confirming ocular tuberculosis [12].

The favorable response to antituberculosis chemotherapy in this case supported the causative connection. This is in accordance with the existing assumption that favorable response to antituberculosis chemotherapy may serve as indirect evidence of disease [13].

The patient outcome underscores the efficacy of systemic antimycobacterial treatment in the setting of tuberculous scleritis and episcleritis. Anterior segment OCT can be utilized in order to assess the extent of the outer ocular wall inflammation and evaluate the treatment response.
